# Impact of artificial intelligence in breast cancer screening with mammography

**DOI:** 10.1007/s12282-022-01375-9

**Published:** 2022-06-28

**Authors:** Lan-Anh Dang, Emmanuel Chazard, Edouard Poncelet, Teodora Serb, Aniela Rusu, Xavier Pauwels, Clémence Parsy, Thibault Poclet, Hugo Cauliez, Constance Engelaere, Guillaume Ramette, Charlotte Brienne, Sofiane Dujardin, Nicolas Laurent

**Affiliations:** 1 Department of Radiology, Valenciennes Hospital Center, 114 Avenue Desandrouin, 59300 Valenciennes, France; 2 Department of Public Health, EA 2694, Lille University, 1 Place de Verdun, 59045 Lille Cedex, France

**Keywords:** Artificial intelligence, Breast cancer, Mammography, BI-RADS classification

## Abstract

**Objectives:**

To demonstrate that radiologists, with the help of artificial intelligence (AI), are able to better classify screening mammograms into the correct breast imaging reporting and data system (BI-RADS) category, and as a secondary objective, to explore the impact of AI on cancer detection and mammogram interpretation time.

**Methods:**

A multi-reader, multi-case study with cross-over design, was performed, including 314 mammograms. Twelve radiologists interpreted the examinations in two sessions delayed by a 4 weeks wash-out period with and without AI support. For each breast of each mammogram, they had to mark the most suspicious lesion (if any) and assign it with a forced BI-RADS category and a level of suspicion or “continuous BI-RADS 100”.

Cohen’s kappa correlation coefficient evaluating the inter-observer agreement for BI-RADS category per breast, and the area under the receiver operating characteristic curve (AUC), were used as metrics and analyzed.

**Results:**

On average, the quadratic kappa coefficient increased significantly when using AI for all readers [*κ* = 0.549, 95% CI (0.528–0.571) without AI and *κ* = 0.626, 95% CI (0.607–0.6455) with AI]. AUC was significantly improved when using AI (0.74 vs 0.77, *p* = 0.004). Reading time was not significantly affected for all readers (106 s without AI and vs 102 s with AI; *p* = 0.754).

**Conclusions:**

When using AI, radiologists were able to better assign mammograms with the correct BI-RADS category without slowing down the interpretation time.

## Key Points

**Table:** 

AI helps radiologists to assign mammograms with the correct BI-RADS category in breast cancer screening.
Using the AI support does not slow down the interpretation time.

## Introduction

In France, a breast cancer screening program exists for women from 50 to 74 years old without symptoms or family history of breast cancer. If an abnormality is detected, an immediate diagnostic assessment is performed. If the mammogram is assessed as normal, images are sent for second reading to an expert radiologist that can detect up to 9% of cancers.

However, these screening programs are regularly debated due to false positives results leading to unnecessary biopsies, overdiagnosis of non-evolutive cancers leading to unnecessary treatments, psychological impact, and induced X-ray dose [1]. Nevertheless, breast cancer screening program in France has shown a decrease of advanced status cancers [2] leading to a favorable benefit/risk ratio for patients [3].

The use of computer-assisted detection (CAD) tools in mammography for breast cancer screening has been widely studied in the past twenty years, particularly in the United States but abandoned due to the lack of improvement in radiologists’ performance [4, 5].

In recent years, artificial intelligence (AI) is booming, thanks to the appearance of Deep Learning (DL) and Convolutional Neural Networks (CNN) techniques which have led to the development of detection and diagnosis assistance systems with performance superior to those of previously available CAD tools [6].

Numerous applications of AI in breast imaging are currently under development, such as cancer detection for different imaging modalities [7–10], triage [11–14], optimization of acquisition protocols and individual risk prediction.

Concerning mammography, a study has shown a very good agreement on breast density between senior radiologists, junior radiologists and the AI software on 2D and synthetic mammography [15].

Moreover, Rodriguez-Ruiz et al., Pacilè et al. and Watanabe et al. demonstrated an increase in radiologists’ cancer detection performance [16] when using AI without slowing down the reading time [17, 18]. The use of AI support in screening with tomosynthesis images was found as well to improve cancer detection and on the other hand, reduced reading time [19, 20]. These studies only evaluate the rate of cancer detection by measuring sensitivity and specificity, not the BI-RADS categorization.

Breast cancer is indeed an interesting application as it represents an important public health matter, with more than 58,000 cancers diagnosed in France in 2019 and more than 12,000 deaths per year.

The procedure to be followed is based on the BI-RADS (Breast Imaging-Reporting and Data System) [21]: for 1 and 2 categories, mammograms are sent to second reading system; if a BI-RADS 3 is assigned, a close monitoring is suggested while for categories 4 and 5 a biopsy is performed.

Therefore, the challenge in organized screening programs is to correctly categorize the examinations to determine the right path to be followed.

Our primary hypothesis is that radiologists are better at classifying mammograms into BI-RADS categories when using AI. The secondary objectives are to evaluate kappa coefficient in 3 categories BI-RADS, radiologist’s performances without and with AI in terms of AUC, and the impact of AI on mammography interpretation times.

## Materials and methods

### Data selection and sample size

Data have been retrospective collected from June 2012 to March 2020 at the Valenciennes Hospital (France). Only screening exams were included in the study (i.e., women between 50 and 74 years old, asymptomatic, without personal, familial breast cancer or breast surgery history, and genetic risk factor). Mammograms were acquired with Hologic Selenia^®^ 3D Dimension^®^ system (which is compatible with the AI system) and have been anonymized.

Mammograms and the medical record of the patients were reviewed by an expert radiologist (13 years of experience in breast imaging, 4 years’ experience as second reader in French organized screening program), to verify the inclusion criteria.

The expert assigned a forced BI-RADS category (hereafter referred to as “GS” (Gold Standard)) solely based on the 4 standard views mammography and mark the position of the most suspicious lesion (if any). Consequently, for cancer cases visible only on complementary images (magnified views, tomosynthesis), he modified the originally assigned BI-RADS to put himself in the same reading conditions as the radiologists who did the reading sessions (at the risk of under-categorizing the lesion).

Of the 397 examinations in the initial dataset, 329 met the inclusion criteria. Of these, 15 benign cases were randomly excluded to reach the target number of 314 examinations to be included in the study (Table 1). The sample was enriched with cancer cases, but readers were not aware of the proportion. Bifocal cancers were excluded.

**Table 1:** BI-RADS distribution of the included dataset validated by the expert radiologist (GS)

		Left	Right	Total
Positive cases	BI-RADS 1	1	1	2 (0,3%)
	BI-RADS 2	5	2	7 (1,1%)
	BI-RADS 3	9	2	11 (1,8%)
	BI-RADS 4	29	25	54 (8,6%)
	BI-RADS 5	28	26	54 (8,6%)
	Total	72	56	128 (20,4%)
Negative cases	BI-RADS 1	89	92	181 (28,8%)
	BI-RADS 2	131	142	273 (43,5%)
	BI-RADS 3	18	21	39 (6,2%)
	BI-RADS 4	4	3	7 (1,1%)
	BI-RADS 5	0	0	0 (0%)
	Total	242	258	500 (79,6%)
Total		314	314	628

Two definitions of *ground truth* have been used:

- A standard definition, i.e., cancer cases confirmed by a positive biopsy result and cancer negative verified by a negative follow-up.
- An expert-based definition, i.e., the BI-RADS classification assigned by the expert radiologist during the including phase (GS).

### AI system

The AI software used in this study is Mammoscreen™ v.1.2 created by the French company Therapixel. This software was designed to detect areas suspected of containing breast cancer, to assess their degree of suspicion on 2D digital mammograms.

The system takes as input the cranio-caudal (CC) and mediolateral oblique (MLO) for each breast and provides as output the position of the detected lesions with a suspicion score for each of them ranging from 1 (benign) to 10 (suspicious) by generating a visual report summarizing the results of the algorithm. The more the score tends towards the extremes (1 or 10), the more the prediction is sure.

The system has been validated for 2D mammography [22] and received Food and Drug Administration (FDA) approval in 2020, as well as CE marking in January 2021.

### Study design

The study was a multi-reader multi-case investigation with cross-over design. There have been two reading sessions delayed by a 4-week wash-out period. During each session half of the dataset has been read with the AI support [Assisted Radiologist (AR)) and the other half without (Non-Assisted Radiologist (NAR)]. Twelve radiologists were involved in the study: 8 radiologists with more than 3 years of experience (hereafter referred to as “senior”) and 4 radiologists with average experience of 1 year maximum in mammography interpretation (hereafter referred to as “junior”). Reading order was randomized among participants. No additional information such as additional images or information about the patient were available to the readers. Previously acquired mammograms were available for 85 exams out of 314.

Reading time was automatically measured for each case. Readers were aware of the time measurement but blinded to the actual measure.

A training on the AI tool and its functioning has been carried out before the beginning of the study.

### Reporting

Readers used the usual reading console of the radiology department (5MP screen manufactured by Barco).

For each case, readers were asked to:

- Mark the most suspicious lesion per breast on both CC and MLO view (when possible)
- Assign a forced BI-RADS score (1–5) for each lesion.
- Assign a level of suspicion or “continuous BI-RADS 100” defined as follows: a scale ranging from 1 to 100 (1–20 for BI-RADS 1, 21–40 for BI-RADS 2, 41–60 for BI-RADS 3, 61–80 for BI-RADS 4 and 81–100 for BI-RADS 5)

For exams read with the help of AI, the AI interface was displayed on the reporting console and synchronized with the reading console. In such way, radiologists could check the suspicion score assigned by the AI before reporting their evaluation.

When analyzing the results, if readers marked a lesion that was not the correct one (i.e., the mark was beyond 1.5 cm from the center of the lesion marked by the expert radiologist during the reviewing phase), the case was considered as misclassified.

### Statistical analysis

Sample size has been calculated using the “kappaSize” R package [23] Sample size estimation information has been provided to determine the number of subjects that are required to test the hypothesis H0: *κ* = *κ*0 vs. H1: *κ* = *κ*1, at two-sided significance level of 5%, with power of 80%, assuming that the outcome is multinomial with five levels. A minimum of 314 subjects was required to demonstrate an effect on *κ* of 0.1.

Results are given in terms of Fleiss quadratic kappa correlation coefficient [24] along with their 95% confidence interval (95% CI). The kappa coefficient has been interpreted as follows:

- 0–0.4 indicates poor association
- 0.4–0.75 indicates medium association
-  > 0.75 indicates high association between the two raters

As there is an inter-observer variability concerning the classification of exams into the BI-RADS 1 and 2 categories as well as into BI-RADS 4 and 5, which both indicate a probably benign and probably malignant examination, respectively, we carried out a second analysis (“3-Cat.BI-RADS”) on 3 categories by grouping BI-RADS 1 + 2, BI-RADS 3 and BI-RADS 4 + 5.

Secondary endpoints (Quadratic kappa correlation coefficient “3-Cat.BI-RADS”, AUC drawn using the “continuous BI-RADS 100” scale and reading time) have been analyzed with statistical methods for reader studies [25].

Statistics tests were bi-sided and considered significant when *p* < 0.05.

Data analysis was performed in R [26] and NCSS 2021 [27].

For all analyses, the statistical individual is the breast, i.e., 2 breasts per patient, and the most suspicious lesion is described.

## Results

### Primary endpoint: impact on agreement between readers (NAR and AR) and the expert

#### Five-category analysis

Results of the evaluations per breast done by the 12 NAR and AR are reported on Table 2 (i.e., 314 × 2 × 12 = 7536).

**Table 2:** BI-RADS per breast assigned by NAR (Non-Assisted Radiologists) and AR (Assisted Radiologists) and quadratic kappa

NAR								Quadratic kappa
BI-RADS readers								
BI-RADS expert (GS)		BI-RADS 1	BI-RADS 2	BI-RADS 3	BI-RADS 4	BI-RADS 5	Total	0.549 95% CI [0.528; 0.571]
	BI-RADS 1*	1255 (57%)	636	198	103	4	2196	
	BI-RADS 2*	801	1819 (54%)	500	238	2	3360	
	BI-RADS 3*	212	167	156 (26%)	62	3	600	
	BI-RADS 4*	196	111	102	260 (35%)	63	732	
	BI-RADS 5*	62	28	37	169	352 (54%)	648	
	Total	2526	2762	993	832	424	7536	

AR								
BI-RADS readers								
BI-RADS expert (GS)		BI-RADS 1	BI-RADS 2	BI-RADS 3	BI-RADS 4	BI-RADS 5	Total	0.626 95% CI [0.607; 0.645]
	BI-RADS 1*	1357 (61%)	586	198	53	2	2196	
	BI-RADS 2*	819	1901 (57%)	465	172	3	3360	
	BI-RADS 3*	174	178	171 (29%)	68	9	600	
	BI-RADS 4*	178	98	98	276 (38%)	82	732	
	BI-RADS 5*	41	9	35	198	365 (56%)	648	
	Total	2569	2772	967	767	461	7536	

* refers to the BI-RADS scores attributed by the expert; to distinguish from BI-RADS scores attributed by readers

We considered the kappa coefficient with a quadratic weighting, for which a deviation of a single step is given a weight of 1, a deviation of 2 steps is given a weight of 2^2^ and so on. This is a severe weighting meaning that it penalizes large deviation very strongly.

The kappa correlation coefficient between the readers and the expert increased significantly from 0.549 [0.528; 0.571] for NAR to 0.626 [0.607; 0.645] for AR. (Table 2).

### Secondary endpoints: kappa “3-Cat.BI-RADS” and subgroups based on readers’ experience, ROC, sensitivity, specificity, and reading time

#### Three-category analysis “3-Cat.BI-RADS”

We carried out a second analysis grouping categories BI-RADS 1 + 2, and BI-RADS 4 + 5.

The quadratic kappa coefficient also increased significantly from 0.528 for NAR to 0.614 to AR (Table 3).

**Table 3:** 3-Cat.BI-RADS per breast assigned by NAR and AR, quadratic kappa

NAR						Quadratic kappa
BI-RADS readers						
BI-RADS expert (GS)		BI-RADS 1–2	BI-RADS 3	BI-RADS 4–5	Total	0.528 95% CI [0.505; 0.550]
	BI-RADS 1–2*	4511 (81%)	698	347	5556	
	BI-RADS 3*	379	156 (26%)	65	600	
	BI-RADS 4–5*	397	139	844 (61%)	1380	
	Total	5287	993	1256	7536	

AR						
BI-RADS readers						
BI-RADS expert (GS)		BI-RADS 1–2	BI-RADS 3	BI-RADS 4–5	Total	0.614 95% CI [0.594; 0.635]
	BI-RADS 1–2 *	4663 (84%)	663	230	5556	
	BI-RADS 3*	352	171 (29%)	77	600	
	BI-RADS 4–5*	326	133	921 (67%)	1380	
	Total	5341	967	1228	7536	

* refers to the BI-RADS scores attributed by the expert; to distinguish from BI-RADS scores attributed by readers

#### Subgroups based on readers’ experience

When evaluating the agreement on subgroups based on readers experience, the kappa coefficient had significantly increased with the help of AI for both subgroups (Table 4).

**Table 4:** Quadratic kappa coefficient NAR and AR per subgroups of readers based on experience

Juniors		
	NAR	AR
Quadratic kappa	0.506, 95% CI [0.466; 0.546]	0.611, 95% CI [0.575; 0.647]

Seniors		
	NAR	AR
Quadratic kappa	0.538, 95% CI [0.511; 0.566]	0.611, 95% CI [0.585; 0.637]

The performances were calculated considering the histology as ground truth, either biopsy-proven cancer for positive cases, or negative follow-up examination for negative cases. The test was considered positive if the examination was classified by a BI-RADS greater than or equal to 3.

#### ROC performance (drawn using the continuous “BI-RADS 100 scale”)

**Fig. 1:**
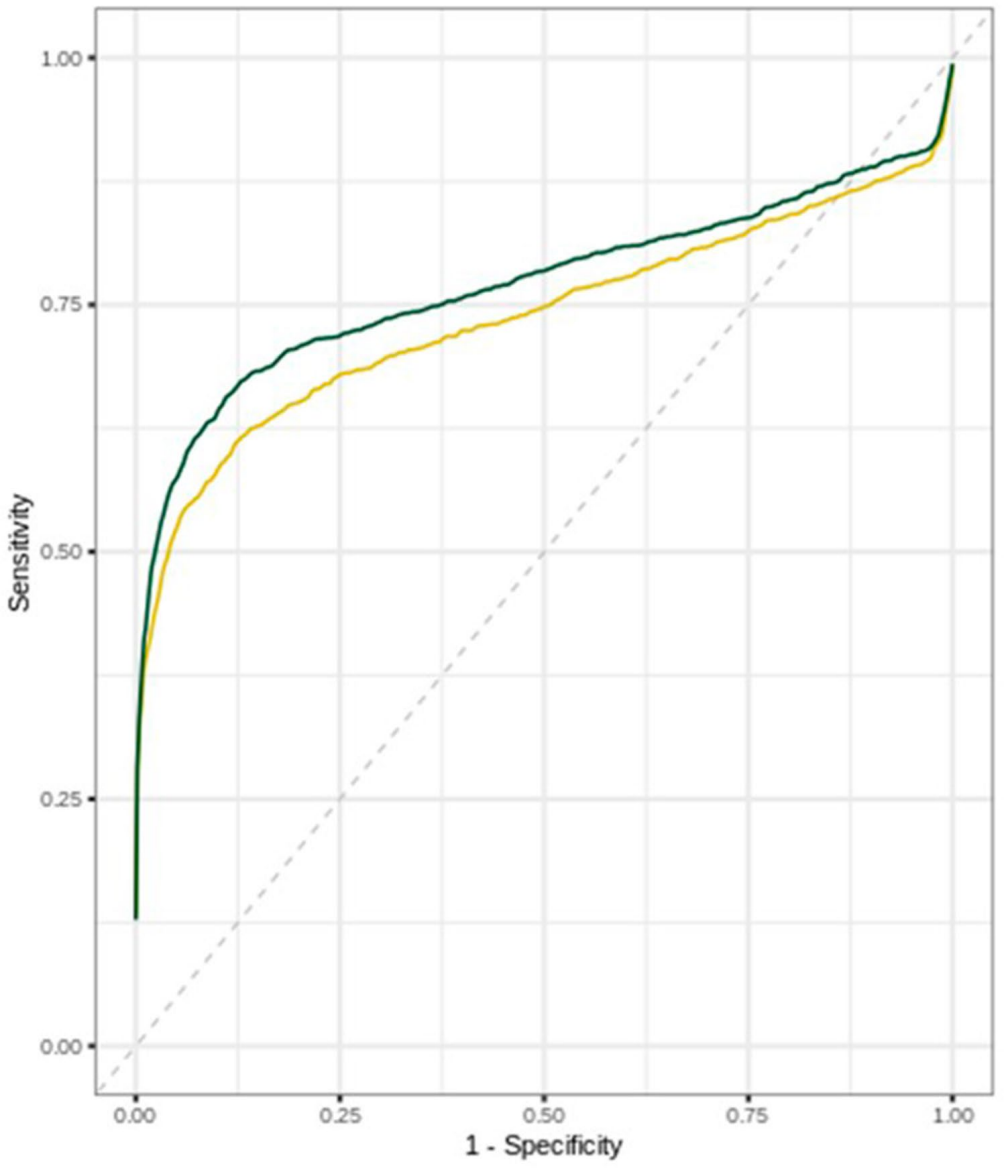
Average ROC curve among readers NAR (yellow curve) and AR (green curve)

On average, radiologists significantly increased their performance in terms of detection with the help of AI, with mean AUC increasing from 0.739 to 0.773 (difference of 0.034; *p* = 0.004) (Table 5, Fig. 1). The same trend was observed analyzing by experience subgroups (Table 5).

**Table 5:** Performances (AUC, sensitivity, specificity), and reading time per reader, NAR and AR

AUC				
Reader	NAR	AR	Δ	
Reader 1	0.747	0.766	0.019	*p* = *0.022*
Reader 2	0.766	0.787	0.021	*p* = *0.028*
Reader 3	0.703	0.758	0.055	*p* = *0.003*
Reader 4	0.725	0.804	0.079	*p* = *0.000*
Reader 5	0.752	0.782	0.030	*p* = *0.006*
Reader 6	0.783	0.757	− 0.026	*p* = *0.396*
Reader 7	0.746	0.794	0.048	*p* = *0.001*
Reader 8	0.705	0.727	0.022	*p* = *0.014*
Reader 9	0.731	0.768	0.037	*p* = *0.012*
Reader 10	0.747	0.742	− 0.005	*p* = *0.202*
Reader 11	0.720	0.794	0.074	*p* = *0.000*
Reader 12	0.742	0.798	0.056	*p* = *0.000*
Average	0.739 [0.689. 0.789]	0.773 [0.723. 0.823]	0.034 [0.012. 0.056]	*p* = *0.004*
Senior	0.744 [0.694, 0.794]	0.776 [0.726, 0.826]	0.032 [0.001, 0.063]	*p* = *0.043*
Junior	0.729 [0.671, 0.786]	0.768 [0.710, 0.827]	0.039 [0.010, 0.067]	*p* = *0.016*

	Sensitivity			
	NAR	AR	Δ	
Average	0.660 [0.630, 0.700]	0.700 [0.680, 0.720]	0.040 [− 0.0002, 0.080]	*p* = *0.051*
Senior	0.670 [0.610, 0.720]	0.710 [0.680, 0.740]	0.040 [− 0.020, 0.100]	*p* = *0.134*
Junior	0.650 [0.580, 0.720]	0.690 [0.650, 0.720]	0.040 [− 0.030, 0.100]	*p* = *0.227*

	Specificity			
	NAR	AR	Δ	
Average	0.790 [0.740, 0.850]	0.810 [0.770, 0.860]	0.020 [− 0.050, 0.090]	*p* = *0.570*
Senior	0.790 [0.720, 0.870]	0.810 [0.740, 0.880]	0.020 [− 0.080, 0.120]	*p* = *0.728*
Junior	0.800 [0.660, 0.930]	0.820 [0.700, 0.940]	0.030 [− 0.110, 0.170]	*p* = *0.654*

	Reading time			
	NAR	AR	Δ	
Average	106.410 [82.320, 130.520]	101.810 [80.850, 122.760]	− 4.620 [− 34.730, 25.510]	*p* = *0.754*
Seniors	93.970 [66.530, 121.420]	96.170 [67.090, 125.250]	2.200 [− 34.080, 38.480]	*p* = *0.899*
Juniors	131.300 [69.210, 193.390]	113.080 [65.040, 161.110]	− 18.220 [− 73.520, 43.070]	*p* = *0.490*

For two radiologists (one senior and one junior), performance was worst when reading examinations with the AI support with a difference in AUC negative, but not significant (*p* = 0,396 and *p* = 202 for readers 6 and 10 respectively, Table 5).

#### Sensitivity and specificity (for BI-RADS greater than or equal to 3)

For example, using a BI-RADS greater than or equal to 3, we measured on average, that the sensitivity increased from 0.66 to 0.70, at the limit of significance (*p* = 0.051). Subgroup analysis shows a similar trend of improvement in juniors and seniors (Table 5).

On average, there was no significant difference in specificity without or with AI support (Table 5).

#### Reading time

On average, there was no significant difference in mammography interpretation time between reading conditions (NAR and AR) (Table 5).

## Discussion

Considering the 5 BI-RADS categories, radiologists classified better in BI-RADS category for AR. We notice that when grouping in 3 categories (BI-RADS 1 + 2, 3, 4 + 5), the quadratic kappa also increased for AR.

The lack of available prior mammograms for most of the examinations included in the dataset, has probably increased the number of potentially benign BI-RADS 3 cases at the expense of the BI-RADS 2 category, and affected the number of false positives.

Actually, applying the BI-RADS mammography classification, high variability has been observed [28], especially for the BI-RADS 3 category [29, 30], as we can see in our study (Tables 2 and 3).

In addition, it has been observed that the strength of agreement varies widely for different types of mammographic finding, especially for subtle findings such as asymmetries and architectural distortion with a weak agreement [31] which affects BI-RADS categorization.

Moreover, during the validation of the dataset, the expert radiologist had access to all reports and information about the patients but did not have systematically the prior mammogram and validated as BI-RADS 2 some benign lesion described as stable in the patient report. Unfortunately, for technical reasons, prior mammograms could be made available to readers for a small subset only which may explain certain discrepancies between readers and the expert.

Regarding the secondary endpoints, we were able to demonstrate an improvement in performance for AR by measuring the AUC, which significantly increased.

**Fig. 2:**
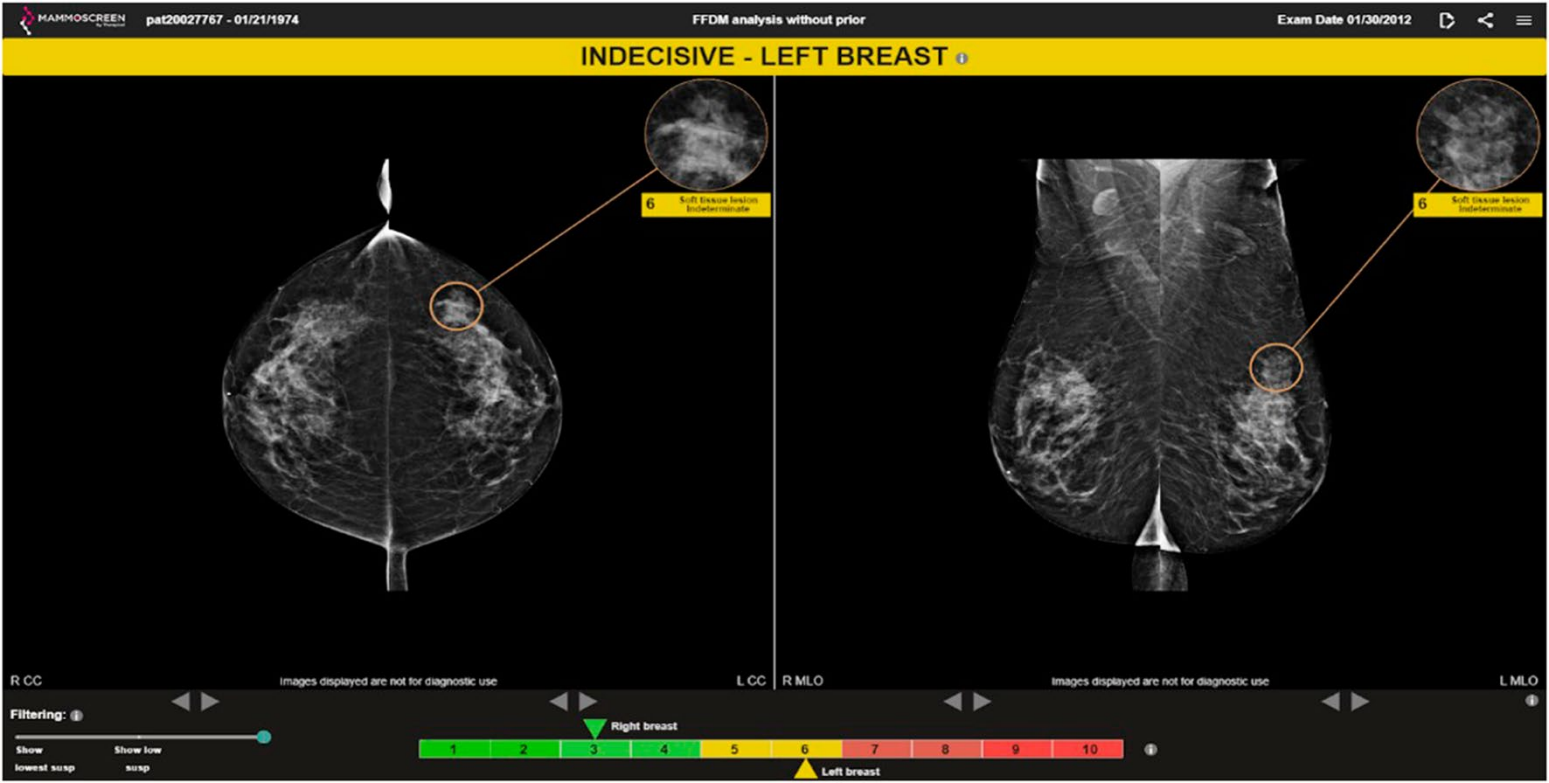
The AI score for this lesion was 6 meaning “indetermined characterization”; without AI, 7 out of 12 readers judged this exam as not suspicious, 2 readers assigned a BI-RADS 3 category, and 3 readers judged the lesion as suspicious for cancer. When reading with AI, one reader only judged the examinations as not suspicious, 2 readers assigned it with a BI-RADS 3 category while 9 readers suspected for cancer

As an example of these findings, the patient in Fig. 2 had a cancer in the left breast. The right breast was cancer free.

Reading time was weakly affected using AI; we noted, however, a time gain of 18.13 s in the subgroup of junior readers.

**Fig. 3:**
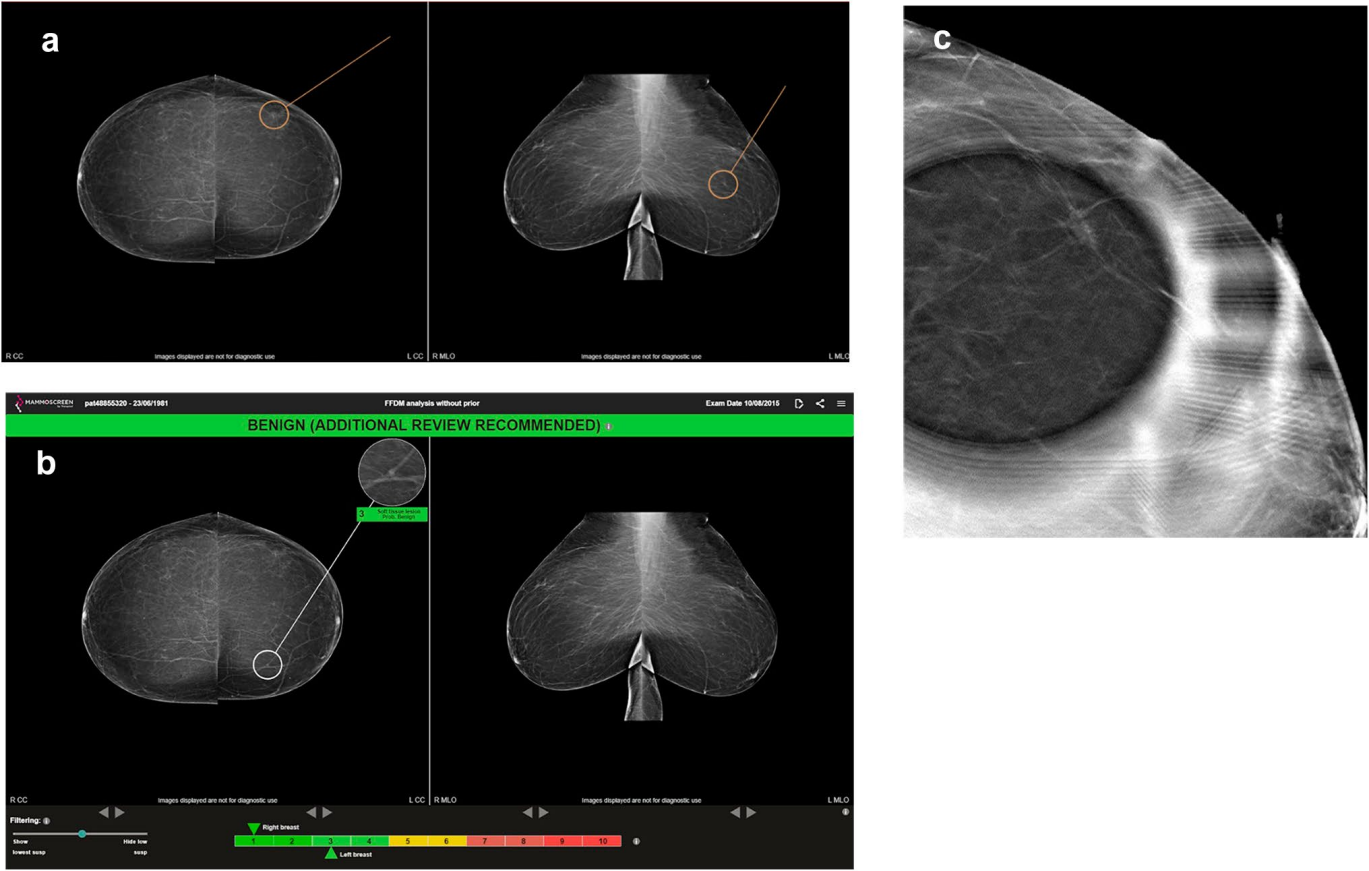
Patient with a cancer on left breast; **a** mark by the expert; **b** mark by the AI; **c** architectural distortion only visible on tomosynthesis

The average sensitivity measured in our study (for BI-RADS greater than or equal to 3) seems to be lower compared to other studies based on the same model such as the one conducted by Rodriguez et al. [17], in which sensitivity and specificity were estimated at 83% and 77%, respectively, when reading without AI support that could be explained by the proportion of cancers classified as BI-RADS 1 or 2 validated by the expert and included in the dataset, particularly for cancers only visible on tomosynthesis images (Fig. 3). In fact, only 2D standard 4-view was made available to the readers while in clinical practice additional images may be considered for the interpretation, notably tomosynthesis images. As an example, for the mammogram in Fig. 3, the cancer was visible on tomosynthesis only. The expert radiologist classified this examination as BI-RADS 2, basing his judgement on a mass on the left breast (Fig. 3a). The AI software did not detect any suspicious lesion except for a benign abnormality in the medial region on the left breast (Fig. 3b). All readers classified this examination as benign (i.e., both breasts were assigned with a BI-RADS 1 or 2). A cancer was actually present in the left breast but visible on tomosynthesis only (Fig. 3c). This example shows that AI cannot compensate for the additional imaging performed in clinical practice, particularly tomosynthesis.

Our sample was enriched with more than 20% of cancer cases whereas the natural prevalence is estimated between 7 and 8 per 1000. In addition, there were 11 out of 50 cancerous lesions among BI-RADS 3 (22%), whereas in clinical practice only 3% of them are malignant [21].

In this type of retrospective study, the reading conditions are far different from clinical practice; the number of examinations read, the reading rate, and the kind of sample are not the same which generate several biases. Gur et al. conducted a study on the laboratory effect by comparing the performance of radiologists in interpreting screening mammograms in clinic with the performance in reading the same examination in laboratory conditions and showed the performance of radiologists was significantly better in clinical conditions [32].

The study in 2020 concerning the same AI tool used in this study, evaluated the performance of radiologists helped by the AI per examination, and showed a better AUC and sensitivity with a decrease in the rate of false negative without affecting readers specificity [18]. Our study demonstrates the interest in using AI in clinical practice for BI-RADS classification of screening mammograms as a help for radiologists, such as it would not be thinkable to replace them, while confirming the improvement in AUC considering a per-lesion analysis.

Our study has several limitations. First, the reference BI-RADS was based on the review of one single expert radiologist. Second, most examinations had no prior mammograms available to the readers. Moreover, readers were using the AI tool for the first time which could have had an impact on the duration of the first interpretations.

This AI tool have limitations as well: it does not integrate the prior mammograms, tomosynthesis and other clinical information; that could generate false positives and false negatives.

Recently, a meta-analysis published in the British Medical Journal [33] reviewed studies testing 36 AI system and demonstrate that AI was less precise in 94% of case than one radiologist and, on the other hand, were not enough specific to replace double reading in screening programs.

However, AI tools should be used as a help for radiologist; a recent retrospective study showed that AI system could reduce workload up to 70% without reducing cancer detection in breast cancer screening with digital mammography and digital breast tomosynthesis [14]

These types of retrospective “laboratory” studies cannot represent performance levels or inter-reader variability during clinical interpretations of the same set of mammograms in a usual work setting. A review of the literature showed that the latest AI models reported good accuracy for breast cancer detection, keeping, however, methodological biases and weaknesses in the test data limiting its application in a clinical screening setting, needing to be resolved in order to be able to extend AI to large-scale population screening [34]. It would therefore be necessary to reproduce this same study prospectively in clinical conditions to validate these results.

A European survey on radiologists’ opinion on AI, published in February 2021, showed that their perception would influence the adoption of AI in clinical practice and highlights that limited levels of AI-specific knowledge are associated with fear, while intermediate and advanced levels knowledge are associated with a positive attitude towards AI. Additional training could, therefore, favor the adoption of such tool into clinical practice [35].

On the other hand, women seem not to support a fully independent use of AI system without involving a radiologist as it was shown in a population survey in a Dutch population in 2020 [36].

AI holds an important potential for transforming the practice of radiology and must excel to become clinically viable; but many parameters have to be considered to measure the cost effectiveness of this tool in breast screening [37].

In conclusion, the results indicate that classified mammograms into BI-RADS categories is obtained with better agreement with the expert radiologist when reading with the support of AI. This can help improve the AUC without significantly increasing reading times.

## Abbreviations

AI: Artificial intelligence
AR: Assisted radiologist
AUC: Area under curve
BI-RADS: Breast imaging reporting and data system
CAD: Computer-aided detection
CI: Confidence interval
DL: Deep learning
FDA: Food and drug administration
GS: Gold standard
NAR: Non-assisted radiologist
ROC: Receiver operating characteristic
